# A thematic analysis of patients’ experiences of receiving treatment for Neovascular age-related macular degeneration (nAMD)

**DOI:** 10.1038/s41433-025-03915-x

**Published:** 2025-07-15

**Authors:** Julie Hapeshi, Samantha Hughes, Martin McKibbin, Peter H. Scanlon

**Affiliations:** 1 Gloucestershire Retinal Research Group (GRRG), Cheltenham General Hospital, Cheltenham, UK; 2 University of Gloucestershire, School of Education and Sciences, Cheltenham, UK; 3 Leeds Teaching Hospitals NHS Trust, Leeds, UK; 4 The Royal College of Ophthalmologists’ National Ophthalmology Audit, Leeds, UK; 5 Nuffield Department of Clinical Neuroscience, University of Oxford, Oxford, UK

**Keywords:** Quality of life, Eye manifestations

## Abstract

**Background/Objectives:**

A positive patient experience is a key element of quality healthcare but data on the patient experience is rarely collected as part of routine care. This study analysed survey and discussion group comments from patients treated for neovascular AMD (nAMD) treatment to identify key elements of the patient experience.

**Subjects/Methods:**

Free text comments from an online Macular Society member survey and quotes from subsequent, local, in-person patient discussion groups were reviewed. Thematic analysis was used to code the responses and to identify major themes.

**Results:**

Analysis include 167 free text comments from Macular Society members and quotes from 2 local discussion groups, with patients and their carers. Key themes, important for the patient experience, included: time and availability of appointments, accessibility and duration of clinic visits, the delivery and quality of care, communication and access to written information and confidence in the healthcare team.

**Conclusions:**

Feedback from patients with nAMD and their carers identified a number of key themes that are associated with either a positive or negative patient experience. Construction of an appropriate patient-reported experience measure and collection of data, either locally or nationally, would help identify areas of the care pathway where improvements may be necessary. Improving the patient experience may encourage adherence to and persistence with treatment and the outcomes.

## Introduction

Patient experience is one of the foundations of quality in healthcare along with the effectiveness and safety of the service [1–4]. However, it is not routinely assessed in many areas of health and social care provision. This may be attributed, in part, to the challenges in implementing and incorporating the assessment of patient experience into routine care. Nevertheless, measuring patient experience could lead to service re-design and, in doing so, improve the patients’ experience and their adherence to treatment; both of which are associated with better outcomes [2]. Further, in the long term, this could decrease service demands and free up increasingly overstretched resources.

Neovascular age-related macular degeneration (nAMD), also called “wet” AMD, is the most common cause of certifiable visual impairment in the developed world [5]. The Royal College of Ophthalmologists (RCOphth) reported in 2020 that AMD affects an estimated 645,000 people in the UK, with this figure expected to rise to 1.2 million by 2050 [6]. nAMD is a chronic condition and the management has parallels with other chronic diseases requiring lifelong care. The treatment is not a single procedure but requires ongoing, timely assessment and/or maintenance therapy with injections into the vitreous cavity of the eye to stabilise or improve vision and a significant commitment to the care plan by the patient and their carers [7]. Both non-adherence to and non-persistence with treatment are known to lead to poor visual outcomes [8–10].

This project aims to evaluate patients’ experiences of treatment for nAMD and, in doing so, hopes to contribute initial understanding towards the development of a nAMD-specific patient-reported experience measure (PREM).

The RCOphth published the first UK National Ophthalmology Database (NOD) audit report of AMD treatment in February 2023 [11] using routinely collected clinical data from electronic patient records of patients receiving treatment for wet AMD and is repeated annually with the third annual report scheduled for publication in Spring 2025. Outcome measures enable an evaluation of services in each centre delivering treatment for nAMD and provide information to support improvements and reduce variation in service provision and treatment outcomes. No measures of patients’ experiences are collected or reported, even though these are vital for the assessment of the quality of the broader aspects of care, and the focus is solely on clinical and safety measures [12].

The Macular Society surveyed its members in 2020 (Macular Society 2020) to understand the effect of injections on them. Responses from almost half of the eligible respondents indicated they had been receiving injections for more than three years, illustrating the long-term commitment to treatment by patients and their families. Following this, in January 2021, a webinar was facilitated by the Macular Society to enable the clinical lead for the UK AMD Audit lead to introduce the concept of the national audit to Society members. The webinar initiated a discussion to help develop a list of potential process and outcome measures for the audit. Following the webinar, members were invited by email to participate in an online survey in which they were asked to choose the 5 most important items from a list of 9 possible process and outcome measures that could be helpful in identifying a high-quality service from a patient perspective. The choice was prompted by the question: “Imagine you are about to start your treatment for wet AMD at a new clinic. Which of the following would be the most helpful to help you choose a “good” clinic? (Pick up to 5)” An additional question asked, “What other aspects of AMD treatment would you like to see included in the audit?” Space was also provided for free text comments. It is these anonymised comments that were analysed and are discussed in this paper.

In the ranking exercise, four items were chosen as being helpful in identifying a “good” clinic, including offering treatment quickly (defined as within 14 days of referral) when first diagnosed (83.8%), confirming the next appointment date at least 2 weeks beforehand (57.4%), providing same day assessment and treatment (52.3%) and the percentage of eyes with vision good enough to drive after treatment (51.7%).

The current paper considers the free-text comments from the online survey along with local patients’ views from additional discussion groups (DGs) to provide a starting point for the development of a Patient Reported Experience Measure (PREM) for patients receiving intravitreal injections for nAMD and other retinal diseases.

## Subjects and methods

The qualitative data were obtained from free-text comments in the Macular Society member survey in April 2021 [13]. These were considered alongside the findings from DGs that were held with patients who have nAMD and their carers.

The DG participants were self-selected and receiving treatment for nAMD by the local NHS Trust. They were contacted via a newly created Patient and Public Involvement Group set up to support the development of a research grant application. A pilot DG of patients and their carers was held in July 2022 to informally explore their experiences of living with AMD and attending for treatment. Information from this was used to formalise the questions for the two main DGs, which were held in October 2022, one in Gloucester and one in Cheltenham, UK.

## Results

Thematic analysis [14, 15] was initially conducted independently on the free-text comments from the questionnaire by JH and SH. Thirteen initial codes were identified and those that were conceptually similar or related in meaning were incorporated to represent five major themes [16] described below. These illustrated overarching areas of importance to the patients and were used to guide the DG conversations. The aim was to explore in greater depth the areas of interest from the Macular Society survey and to establish any additional concerns that needed to be discussed. The themes included:

1. “***The availability, timing [and] choice of appointments***” - including scheduling and changing appointments, and contacting the booking office.
2. **Challenges associated with** ***“time spent at [and accessibility] of clinics”*** - mainly the variable length of time spent in the clinic and the burden on carers, managing other health conditions.
3. ***“Quality of care”*** **around receiving treatment** – concerns about inconsistencies in approach by staff, not being listen to or treated as an individual, seeing the consultant at least annually.
4. **The need to be** ***“kept up to date on treatment”*** – desire for clear communication, including up to date written information especially at the start of treatment.
5. ***“Knowing the expertise of [and] having confidence in the people giving the treatment”*** – expressions of lack of confidence in some staff.

The DGs captured the views of six patients and three carers and each patient was given a short questionnaire to elicit some demographic information. All patients were over 65 years with the oldest being over 90 years old. They had been living with nAMD for periods of time ranging from less than one year to more than 15 years and half of the group attendees lived alone. Half were still driving although one said this was “only during daylight” and one person was still in paid employment.

The initial themes identified from the free-text comments collected via the Macular Society survey were utilised as prompts to facilitate the DGs and to elicit whether any similarities or differences to those observed in the data from the members survey were expressed.

The responses from the DGs (G1 or G2) were subjected to thematic analysis and are discussed in conjunction with the findings from the members survey below. Some quotes are included in the body of the results section and other illustrative quotes are reported as supplementary data with the source of the quote identified as from a survey respondent or discussion group attendee.

### “The availability, timing [and] choice of appointments”

The patients’ suggested that their experience was or would have been enhanced if the appointment for their next clinic was given on the day of their treatment or by post soon after their appointment (quote 1). Patients felt that this enabled them to schedule their treatment into their lives, to have holidays and allow for some flexibility without missing treatments.

Conversely, delays in receipt of their next appointment, or a need for them to phone to chase their appointment, was anxiety provoking and resulted in adverse treatment experiences (quote 2).

Worry about missing an appointment or delays in their next review were universal concerns voiced by the patients in the DGs. Attempting to address this concern may help to enhance the patients’ nAMD treatment experience. More specifically, one patient suggested that being able to attain *“follow up appointments within the timeframe indicated by the consultant” (Survey respondent 13)* would give them reassurance in their eye health and confidence in their care. Patients commented that their appointments become especially problematic when having nAMD treatment alongside treatment for additional conditions such as cancer. These situations lead to missed treatments and deterioration of their vision (quote 3).

There were concerns about the impact of delays or missed appointments on their eye care as well as on their overall health and wellbeing. Providing information with regards to dealing with missed appointments would hopefully offer patients some reassurance and thus enhance their nAMD treatment experience.

In quote 3, one patient highlights how her other health problems resulted in delays in her nAMD treatment and ultimately resulted in the deterioration of her eye health. This was not an isolated case, several other patients stated how various competing health needs such as poor mobility and deafness, glaucoma and dementia had affected their nAMD treatment experience and their overall eye health.

Patients proposed that having a direct and dedicated phone number to call the injection clinics rather than a line to a general booking office would be helpful if changes to their appointment needed to be made (quote 4). They felt that talking to people who understand the importance of the scheduling of injections is crucial when dealing with appointment changes. Changing appointments is possible but does rely on knowing who to contact (quote 5).

### Challenges associated with “time spent at [and accessibility] of clinics”

Many patients considered it to be reasonable to expect that a treatment appointment could be completed within two hours and, ideally, in less than an hour. Completion of their appointment within this timeframe made for a good treatment experience, as one patient stated in, this was not always the case (quote 6).

When the duration of their appointment exceeds 2 h, patients reported that they can feel increasingly apprehensive (quote 7). Longer times in clinic were also considered to increase the burden on family members (or carers) who, typically (considering their needs), accompany the patient to their nAMD appointment. A primary issue with regards to family burden was the cost of parking and the stress caused by clinics running late when using “pay and display” parking when tickets might expire.

Patients suggested that support with parking, in the same way that cancer patients have financial concessions or disability permits issued during their chemotherapy, would help to improve their care experience. One patient who had received treatment for cancer observed how she was given a voucher for free parking during her cancer treatment but she does not receive this for her nAMD treatment where the appointments are just as frequent and longer in duration.

It was also expressed that the offer of benefits such as free parking or disability permits would help immensely (quote 8). Participants suggested that a quick turnaround in clinic, taking no more than one hour, would help to reduce the associated stressors. A regular update about waiting times and delays for treatment to allow for carers to plan how long to be “off-site” would also be appreciated, simply knowing how long they were going to be waiting would make life easier (quote 9).

One carer had the solution of dropping the patient off for their appointment and waiting offsite until they were ready to be collected, to avoid the costly parking charges and to prevent their dogs, who accompany them in the car, getting agitated (quote 10). Additionally, travel concerns had an adverse effect on the patients nAMD treatment experiences (quote 11). However, one patient commented that as her eyesight worsens, she is finding it very stressful to use her mobile phone to call to be collected and that finding her way to the rendezvous point is increasingly difficult especially after having injections in both eyes.

A further perception with regards to the service was that it was like a conveyor belt. Patients use of words such as *“cattle” (Survey respondent 36) and “tick box” (Patient, G1)* depicted an impersonal process that they sometimes felt that they were going through while attending clinic. The patients accepted that this was almost inevitable due to the high volume of patients and felt that there may be no other way around the structural process to manage the size of the clinic.

Patients acknowledged that the way the clinic was organised was a bit of a trade-off if waiting time in the clinic was to be kept to a minimum (quotes 12 and 13). However, the process-driven way of managing the clinic was not deemed suitable for everyone. Patients felt that the care of people with other morbidities, including dementia would benefit from a different approach that improved continuity of care (quote 14).

For the patients, a lack of refreshments, especially in hot weather and when the clinic was delayed was a problem. Before Covid, water was available and some patients mentioned a refreshment service run by volunteers, but this had disappeared and had not restarted since the pandemic, which was seen as a detriment to the patient’s experience.

### *“Quality of care”* around receiving treatment

The patients felt that they should be at the centre of the care that is being given. Receiving timely treatment according to the schedule requested by the consultant was undoubtedly seen as important but the patient perceived that the consideration of them as a person was a primary factor in facilitating their overall treatment experience. Being acknowledged/recognised as a human being was deemed to be an important aspect of the treatment experience. Additionally, having a treatment team that appreciates and accommodates the patient’s broader life needs was seen as a superlative aspect of care (quotes 15-18).

Conversely, quote 19 suggests that on occasions, patients felt as if they were not listened to or as if they did not know what was best for them in terms of their own care. Such experiences were severely stressful for the patients and often required them to take assertive action, which they did not like to do.

As part of their treatment, patients also expressed that they would like to see their consultant for an update on their condition at least annually (quote 20). Whilst the patients appreciated that they did not need to see the specialist at every appointment having an opportunity to have a periodic review with the consultant, to understand scan results and objective assessment of disease progression or stabilisation along with information about new therapies and developments was seen as an important aspect of treatment and decisions about on-going care.

A further point that patients expressed was apparent inconsistencies in the procedures that were employed by the staff. For example, one patient talks about the “drape” and “egg cup” techniques (quote 21) and felt that it would be helpful if the use of different injection techniques – the “drape” vs the “egg cup” (speculum) were fully explained. Patients were not aware as to why different techniques are used; whether there is a clinical reason or whether it is just operator preference. There were also comments about clinical staff who do not wait long enough for the local anaesthetic to work (quote 22), and whether using an egg timer to take the guess work out of the estimate of the timing might be helpful.

Also, another patient (quote 23) alludes to the lack of sufficient time for drops to take effect that may be a consequence of the increasing pressure on the service, ultimately, resulting in increased anxiety and a poorer experience.

### The need to be “kept up to date on treatment”

Everyone agreed that clear, written information at the start of treatment that is readable, with phone numbers and websites (kept up to date) was crucial, including information about Low Vision clinics. Thus, patients expressed the importance of finding a means to keep information, such as emergency contact details updated. On a similar note, patients expressed much concern as to what they need to do, and whether adequate actions are taken, if they experience an emergency with regards to their nAMD. Their thoughts are highlighted in quotes 24 and 25.

### “Knowing the expertise of [and] having confidence in the people giving the treatment”

A number of the patient’s narratives (quotes 26 and 27) in this theme identify particular members of staff that they like to see and the demeanour of staff that they find reassuring.

Other patients expressed doubt as to whether procedures were being correctly followed as part of their treatment resulting in a lack of confidence or doubt in the service. These patients inferred that less able staff contributed to a deterioration in eye health and/or pain in their treatment. An audit of the injection technique was suggested to ensure it is of a good standard. From the DGs patients were able to identify staff who, from their point of view, had injection techniques that caused them pain. This enhanced anxiety in some patients, and may deter others from returning; these views are depicted in the excerpts below:

Staff needed to be aware that listening skills and understanding the patient’s perspective especially when English is not their first language (staff and /or patients) was crucial.

Patients were accepting of many of these issues providing they had a proper explanation.

## Discussion

Data used from an online survey conducted by the Macular Society [13], involving their members who had personal experience of nAMD treatment, helped to identify and explain five keys factors that represent a high-quality service from the patients’ perspective. These factors mainly involved the patient’s perspective and experience of the care pathway across the five key areas: namely appointments, attending the clinic itself, treatment, communication, and the staff. Although there were many positive comments, these were often tempered with suggestions for improvement. For example, with regards to staff, patients generally perceived them as being *“friendly and kind”* (Patient, G2) however, this was typically followed with the concern that they were unable to form a lasting relationship with their care team because “*there’s never any continuity”* (Patient, G1), that is, they purported rarely seeing the same person twice. Consequently, the recommendation was made to promote continuity of care to enable *“a bit of rapport”* (Patient, G2) to develop between them and their treatment team.

The patients’ perspectives from the current study are similar to prior research findings. For example, when exploring care provision [17] reported fifteen dimensions which include the overarching priority of treatment to preserve vision. Additionally, receiving information about their diagnosis and treatment, waiting times in clinic and trusting those giving treatment scored highly as priorities for the patients. A further study [18] which investigated patients experiences of undergoing nAMD treatment, identified three main themes that focussed on the burden of treatment, how to minimise the burden and the importance of treatment. It was also reported that the indirect costs associated with attending treatment appointments such as fuel costs, public transport and car parking when incurred on a monthly basis can be difficult to manage, similarly to that reported by the patients in the current study. Minimising the burden of treatment included coping with the scheduling of appointments, managing pain and the anxiety of receiving the injections. Regardless of the perceived negative aspects of receiving injections, patients recognised the importance of receiving regular care and expressed the motivation to continue treatment in spite of this. Patients who were living with nAMD in the current study adapted to their lives, often with minimal support from their partners, families or friends. There was a desire to not be viewed as a “typical nAMD patient” [19] they desired to be treated as the whole person that they are in spite of their condition.

In an audit of patients lost to follow-up [7], of 1328 patients attending for intravitreal injections over a 5-year period (2014–2018), increasing age was found to be an important factor in ceasing treatment and ill-health was a key reason for non-attendance. Other factors that impacted on adherence and attendance included transport and administrative issues with appointments but few patients stopped treatment due to anxiety or pain linked to their injections. The patients in the current study expressed similar views with considerable concerns mentioned around transportation, accessibility to clinics and difficulties in scheduling appointments. While these were frustrations, they also demonstrated persistence with their care in recognition of the health implications.

The culmination of findings from the current study, as well as those reported in six other publications [18, 20–24], suggests a desire for patients to have enough information to navigate the care system and to minimise the burden associated with regular, frequent injections required for long term. However, there remains little agreement about exactly what to measure and how, in terms of patient experience.

The comments analysed and reported in this paper highlight elements of the care pathway that are important to patients with nAMD. However, such data is not currently collected as part of routine clinical care.

Capturing data on patient experience is an important pillar to providing quality care and is essential if care is to be truly patient-centred. It also has a major role to play in improving patient safety [25].

Improvements in patient experience could help to ease issues that lead to non-adherence and non-persistence with nAMD treatment and improve clinical outcomes [2] but there is currently no validated PREM that is specifically designed for assessing patient experiences of treatment for nAMD. There are measures for capturing satisfaction with treatment but these do not go far enough especially in capturing treatment-related anxiety and treatment burden [18]. A validated PREM developed as a questionnaire that could be used as part of routine care in high-volume AMD injection clinics, without being burdensome to staff or patients, would complement the clinical outcomes of the UK AMD audit and may help ensure that local delivery of a high-volume service retains a patient focus.

The Royal College of Ophthalmologists has chosen to use Cat-PROM5 [26] as a patient-reported outcome for implementation in the National Ophthalmology Database (NOD) National Cataract Audit. Although the nature of the procedure, i.e. not repeated injections, makes this an easier administration process it has nevertheless not been universally implemented.

There is a need to collect and report on the wider experiences of patients undergoing NHS treatment for nAMD. Data gathered from large numbers of patients in national audits has already identified variation in clinical care and highlighted best practice with the aim of improving the quality and outcomes of treatment but the picture is incomplete without information about the experience of patients. To date, there is currently no suitable PREM for use alongside national clinical audit. The responses in this paper provide a strong starting position for the development of a patient experience measure for nAMD.

## Summary

### What was known before

- Very little has been written about to patient experience in treatment clinics for wet AMD.

### What this study adds

- Feedback from patients with nAMD and their carers identified a number of key themes that are associated with either a positive or negative patient experience.

## Supplementary information

Supplementary material to AMD paper Eye 25 -660

## Data Availability

All data supporting the findings of this study are available within the paper and its Supplementary Information.
