# Supplementary material for: A thematic analysis of patients’ experiences of receiving treatment for Neovascular age-related macular degeneration (nAMD)
Source: Eye (Lond). 2025 Jul 15;39(13):2565–9. doi: 10.1038/s41433-025-03915-x (PMC12402154; doi:10.1038/s41433-025-03915-x)
Supplement: Supplementary file 1 — Supplementary material to AMD paper Eye 25 -660 [file 41433_2025_3915_MOESM1_ESM.pdf]

A thematic analysis of patients' experiences of receiving treatment for Neovascular age-related macular degeneration (nAMD).

EYE- 25-660

Supplementary material (to version 4.3)

Key to source of illustrative quotes.

G1 – Discussion Group 1

G2 – Discussion Group 2

Survey Respondent - respondent to survey coordinated by the Macular Society on behalf of the National Ophthalmology Audit lead for AMD.

Quote 1

"I think our appointment system is fantastic. Within 3 or 4 days you have your next appointment sent." (Patient, G1)

"Appointments on the whole [are] brilliant because I have got my November one through now, they always do it…4 weeks and 8 weeks. Absolutely first class, I know exactly when I am going." (Patient, G1)

"I usually get a letter after clinic. It's usually a Tuesday these days and I have a letter by Friday saying when my next appointment is." (Patient, G1)

Quote 2

"One of the most distressing aspects of treatment is not knowing when the next appointment will be and virtually having to beg the appointments staff for an appointment on time or having to get hold of the department secretaries and get them to intervene…" (Survey respondent 22)

"I should have 4 weekly injections but quite often I can't get them [because they are difficult to schedule around my other treatment] and I'm waiting for too long and this eye has now become very damaged because I waited…I've had quite bad health in the last 3 years with breast cancer and then gall bladder problems…and then I've had to have a melanoma and two face operations so I'm just hoping that I am coming out of that because it has been very difficult and what with Covid as well…my left eye is really quite bad now and I am just hoping that my right eye is going to hold out." (Patient, G2)

Quote 3

"It was terrible when…I missed an appointment when I was actually in hospital and I said that I should be at the eye clinic but nothing was followed up and so I totally missed a whole thing [treatment cycle] so I was left for 9 weeks and that is when that eye got really damaged." (Patient, G2)

Quote 4

"You need the number of who to contact. There is a specialist for the, I have forgotten what it is called, for the retinal injections there is a secretary that deals with the clinics with that but…you need a list of who to contact…You need a direct number really rather than having to go through the booking office." (Patient, G1)

Quote 5

“Admin staff were not aware that I was supposed to have injections every four weeks due to poor communication between them and the doctors. I was frequently given late appointment but hated phoning as they took it personally.” (Survey respondent 107)

Quote 6

“The first time I was…in and out within three quarters of an hour, diagnosed, chat with the consultant, injection everything. Second time…took about an hour and a quarter so that was still quite good.” (Patient, G2)

Quote 7

“The time spent at the Clinic could surely be addressed. Sometimes the whole procedure takes 45 minutes, more often, for the same procedure it takes 2-3 hours.” (Survey respondent 1)

I’ve often been waiting up to 4 hours in a clinic. This is very stressful, and not a good practice.” (Survey respondent 160)

Quote 8

“So that is an issue, when people are regularly having to go and park, I mean there are some places when [the patient] had cancer for example you get issued with parking but no such thing seems to apply. It would help.” (Carer, G2)

Quote 9

“From my point of view, I’ve resolved what I can do while I’m waiting but I think it’s the communication as to [the patient] is now in there...how long is she going to be in there? I know we’ve got mobile phones but [the patient] sometimes doesn’t know what’s going on so you’re just waiting. Maybe there ought to be some sort of arrangement to feedback to the patients where they are in this queue, how much longer you are going to be.” (Carer, G2)

Quote 10

“The dogs don’t enjoy sitting in the back of the car in the car park. So, I’ve had to devise a system to cope with the dogs as well as waiting for [the patient] to phone to say that she’s free. So, I’ve got walks that I can do in fine weather and walks that I can do…when it’s raining…because I’ve had to wait 2 hours, 3 hours sometimes it’s quite a time to kill.” (Carer, G2)

Quote 11

“After having the injection, I had to get to the bus stop…and I couldn’t see, I didn’t know where I was going.” (Patient, G1)

“Accessibility of clinics!!-you cannot drive to appointment-yet I have to take 4 buses and 2 trains to make a round trip-2 hours to get to appointment, 4 hours at appointment and 2 hours to get home!!” (Survey respondent 19)

“Waiting for another taxi to home…causes even more upset and expense.” (Survey respondent 65)

Quote 12

“I think you accept it [feeling like a conveyer belt] because it’s the only way it’s going to be...You’ve got to have so many tests done and you just know the routine and you’ve just got to accept it.” (Patient, G1)

Quote 13

“When I arrived, there was no space in the waiting room…so I sat in the corridor and no one was taken out of the waiting room for the first 10 minutes. But in fact, I was only 20 minutes late and I was out in an hour and a quarter.” (Patient, G2)

Quote 14

“Help for patients with other medical problems - e.g., poor mobility (the hospital is huge), also deaf patients (please do not just speak louder). Explain all the processes and tests. Please do not leave people sitting in dread of injections. Explain! For the longer (5 hours) first visit, help to get refreshment. I was not offered even a glass of water!” (Survey respondent 17)

Quote 15

“The personal. I am a person, a whole person, not a picture on the scan screen. It’s happening to Me. Every patient feels that, surely. Alas, one has to feel pitiful gratitude to be treated at all. The whole is run ‘by the science’ and sadly economy. And by the soul-less expediency of ‘outreach’ portacabins in supermarket car parks with no toilets [and] overwhelmed staff. Wonderful nurses. Always wonderful nurses…Four years, both eyes, just a number on the rota.” (Survey respondent 80)

“How you are treated as a person [impacts on your experience], how well they [the treatment provider] explain what is happening with your eyes and eyesight, not just the treatment.” (Survey respondent 27)

Quote 16

“It makes a huge difference to have someone who’s friendly and kind and chatty. You might have someone be extremely efficient but who doesn’t really chat to you makes you more nervous.” (Patient, G2)

Quote 17

“It is very special if I see [name of consultant]. It’s always very special. He is very reassuring and you really feel that he absolutely knows your case…He is so pleasant as well.” (Patient, G2)

Quote 18

“[Name] always said look we will work with you. She’s always said that it is always important that you have your holiday so we will work with you…Very, very good.” (Patient, G1)

Quote 19

“I think sometimes they [the treatment team] need to listen to the patient…I was having an injection and…I’m allergic [to / intolerant of iodine and [the consultant] said we’ll give you the other one [type of drops]. I went in…and the nurse said “I “see you say you’re allergic to iodine” … she said “well you can’t

be allergic to iodine" and I said "well I'm intolerant." So, she did the one eye and I thought what…I can smell iodine and my eye went yellow and I said "I don't have iodine at all." And she said "actually it won't…you'll probably find that it will just get a little bit…" And I said "no" and actually I was a little bit abrupt with her I said "I've sat on the edge of my bed at 2 o'clock in the morning and it's felt like shards of glass in my eyes" and I asked for saline and she did actually wash it out…That was bad. And for the rest of the day, although she washed it out, there was that soreness, it's like shards of glass." (Patient, G1)

Quote 20

"I have been attending a leading clinic for 2.5 years and have not yet seen a consultant and the over worked doctors have no time to discuss any worries with you." (Survey respondent 38)

"Meeting with a consultant at least annually to discuss treatment plan and answer questions as they arise." (Survey respondent 51)

"I would like to see a consultant occasionally at the clinic to get an up-to-date progress on my eyes and the regularity of my treatment he or she prescribes to be adhered to…I don't know when I saw a consultant last nor know who he or she is." (Survey respondent 96)

Quote 21

"They [the staff] do it in different ways…There's one way of doing it where they put this drape that hangs down and then clip your eye open and they fix it to the bottom. And then there's this other thing that they just call the egg cup. That's the smaller one and it's better than the drape because you can feel a little claustrophobic with this drape coming over you and the last time, I went the nurse used the drape on this eye, this side she used the egg cup." (Patient, G2)

Quote 22

"I think after they [the staff] have put the anaesthetic drops in your eyes, when I was first going, there used to be a timer, there would be a signal but they don't do that now…and I wonder whether sometimes the injections are a bit too soon after the drops going in your eye because I mean they're not painless, they are not painless for me, they can be quite painful. Um so I just wonder whether they leave that time. I mean with so many patients to be seen I expect they want to get on as quickly as possible." (Patient, G2)

Quote 23

"How long from instilling anaesthetic eye drops into the eye/s to be injected until the injection is given (I have found that not long enough is given and the anaesthetic hasn't had time to work and some injections are very painful because of it). Sometimes I feel the anaesthetic just starting to work as I am leaving the hospital. How thoroughly should the iodine be flushed out of the eye afterwards? I usually find that some injectors use too little flushing solution (regardless of being asked to do more) which can cause gross discomfort for a few days afterwards." (Survey respondent 91)

Quote 24

"Much more detailed information [is needed] - I have had AMD for over a year and lost sight in one eye, I still know only what I have researched on the internet." (Survey respondent 84)

Quote 25

"If they [the healthcare professionals] have got something they tell you, give you the leaflet at least if you don't absorb everything at that moment you can refer back." (Patient, G1)

"Patient information leaflets I think [would be helpful] but in simple language." (Patient, G1)

"The other thing about communication is the low vision clinic…I don't know anything about the low vision clinic. I don't know whether I should be going or should have gone but I assume by talking to people I know at the clinic I go to that you are referred just it's for using instruments and things to help but you see you didn't know about that…When can you apply for financial help…? No idea." (Patient, G1)

"I was given one [an emergency contact number] many years ago from the hospital but when I rang that one it was no longer in existence." (Patient, G2)

"When I panicked because things had changed, I couldn't get any help, not at all. Not from my optician, the hospital and not from my doctor and I did panic." (Patient, G2)

"My biggest bugbear is the [phone] numbers…you've got so many numbers that you can phone and when you want to get hold of somebody it's the triage number that will only deal with things…your retina etc. I've had a couple of injections where I've had bleeding and all the rest of it, they don't do anything because they say we are only concerned with the back of the eye." (Patient, G1)

Quote 26

"She is excellent, she is the only injector that actually uses a mask and I can go home and my eyes are absolutely fine. Sometimes with other injectors you know you leave and they're quite sore but with her it's absolutely amazing." (Patient, G1)

Quote 27

"Staff who are efficient but not friendly make me nervous." (Patient, G2)

"Staff who are friendly and welcoming, who will hold your hand if necessary and explain everything as they do any procedures." (Survey respondent 18)

"I think people have views on some people that give injections and I question whether some of them should be retrained a little bit." (Carer, G1)
